# Ropivacaine inhibits the proliferation and migration of colorectal cancer cells through ITGB1

**DOI:** 10.1080/21655979.2020.1857120

**Published:** 2020-12-21

**Authors:** Xiao Wang, Tianzuo Li

**Affiliations:** Department of Anesthesiology, Beijing Shijitan Hospital, Capital Medical University, Beijing, China

**Keywords:** colon cancer cells, ropivacaine, ITGB1

## Abstract

To study whether ropivacaine inhibits the proliferation and migration of colon cancer cells through ITGB1 (Integrin beta-1). First, the effect of ropivacaine on cell proliferation and migration was detected by MTT and Transwell. DAPI staining, annexin V staining and Western blot were used to detect the expression of apoptosis-related proteins to investigate the effect of ropivacaine on cell apoptosis. Using bioinformatics software to predict the potential drug targets of ropivacaine. RT-PCR, Western blot and immunofluorescence verify the distribution and expression of the drug target ITGB1, and detect its downstream-related proteins to further prove that ropivacaine affects colon cancer cells by acting on ITGB1 protein. 1. Ropivacaine significantly inhibited the proliferation of colon cancer cells and promoted their apoptosis 2. Ropivacaine could interact with ITGB1 protein, and inhibited the expression of ITGB1 protein in colon cancer cells, thereby affecting its downstream signaling pathway. Ropivacaine regulates the function of colon cancer cells by targeting the expression of ITGB1 protein and affecting the activation of its downstream signaling pathways.

**Abbreviation:** Integrin beta-1 (ITGB1); 3-(45)-dimethylthiahiazo (-z-y1)-35-di- phenytetrazoliumromide (MTT); 4. 6-diamimo-2-phenyl indole (DAPI); Reverse transcrption PCR (RT-PCR); Colorectal cancer (CRC); Local anesthetics (LA); voltage-gated sodium channel (VGSC); dulbecco s modifed eade medium (DMEM); propidium iodide (PI); dodecyl sulf ate, sodium salt-Polyacrylamide gel electrophoresis (SDS-PAGE); Polyvinylidene Fluoride (PVDF); BCL2 associated X (Bax); Focal Adhesion Kinase (FAK); extracellular signal-regulated kmase (ERK); alpha serme threcnime-proteim kinase (AKT); Glyceraldehyde-3-phosphate dehydrogenase (GAPDH); Tris-buffered salme with 0.1% Tween 20 (TBST); Similarty ensemble approach (SEA)

## Graphical abstract

**Figure:**
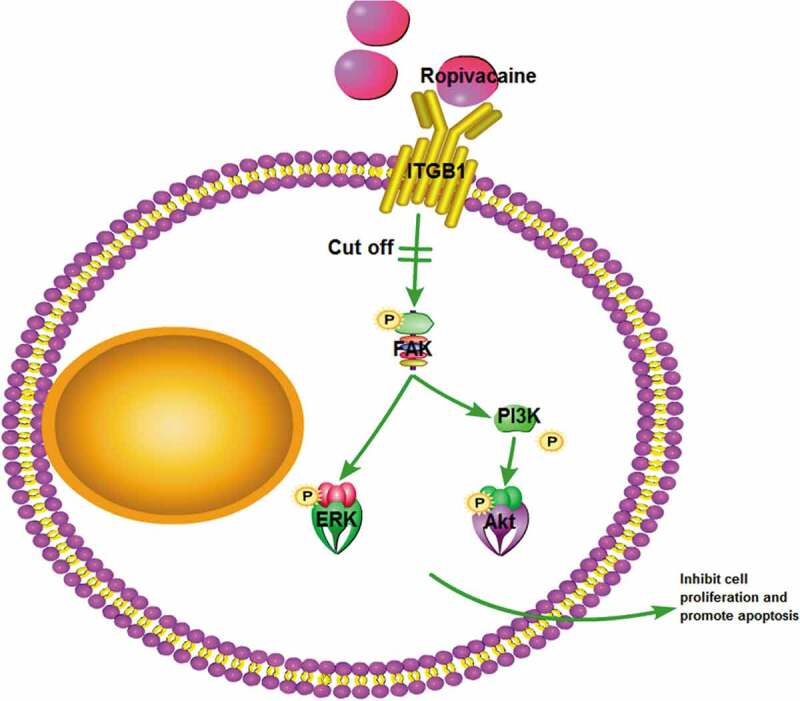

## 1. Introduction

More than 500,000 people die of colorectal cancer every year in the world [1]. Colorectal cancer (CRC) is one of the most common malignant tumors in the world, and its incidence rate and mortality rate have increased rapidly in recent years [2,3]. In China, CRC has become one of the eight most common malignant tumors [4]. The death of patients is caused by the metastasis and spread of cancer cells. Surgical resection is the main treatment for colorectal cancer.

In recent years, more evidence has shown that the risk of cancer spread after surgery has increased [5,6]. Tumor vascular injury and surgery cause cancer cells to enter the blood circulation. In the process of wound healing after operation, the level of growth factor increases, and the balance between pro-angiogenic factor and anti-angiogenic factor is broken, thus stimulating the growth and metastasis of cancer cells. In addition, perioperative immunosuppression would abolish the anti-cancer immune monitoring. This lethal combination releases cancer cells, impaired immune function and high growth factor levels could increase the risk of cancer recurrence during the perioperative period. Some retrospective studies have shown that the use of local anesthesia in cancer surgery can improve survival rate [7,8]. The exact mechanism of local anesthesia may not have been fully elucidated, but it can reduce stress response, and is proposed to prevent postoperative inflammation and immunosuppression [9,10]. Local anesthetics (LA) act on all cells by blocking voltage-gated sodium channels (VGSC), and may also induce apoptosis [11], demethylated DNA [12], blocking the invasion of metastatic cancer cell in vitro [13], and other direct inhibitory effects on cancer cells, and may have direct cytotoxicity [14] and anti-proliferation effects [15]. Integrins are cell membrane receptors and bind to extracellular matrix and participate in a variety of metastases, including tumor angiogenesis; integrin signaling regulates cell apoptosis, cell adhesion, proliferation and migration through gene expression, playing a key role in regulating cell growth and tumor progression [16]. ITGB1 is an integrin, mainly expressed in normal cells and tumor-related cells, regulating angiogenesis, tumor progression, apoptosis and metastasis [17,18].

In this study, we investigated the effects of ropivacaine on the proliferation, migration and apoptosis of colon cancer cells, and further studied whether ropivacaine regulates the biological function of colon cancer cells through ITGB1.

## 2. Materials and methods

### 2.1. Materials

#### 2.1.1. Reagents

Ropivacaine (Beijing inno-chem Co., Ltd.); MTT, Crystal Violet and DAPI were purchased from Beijing Solarbio; Rabbit anti-human ITGB1 antibody (CST, #34,971); goat anti-rabbit IgG-HRP antibody (HuaBio Biotechnology Co., Ltd. article number: HA1001); FITC Annexin V Apoptosis Detection Kit (BD Pharming).

#### 2.1.2. Cell lines

Human colorectal cancer HCT116 cells, SW620 cells were purchased from ZhejiangRuyao Biotechnology, and the cells were cultured in DMEM supplemented with 10% fetal bovine serum and 1% penicillin/streptomycin and incubated at 37°C in a constant temperature culture containing 5% CO_2_.

#### 2.1.3. Instruments

CO_2_ constant temperature incubator (ThermoFisher); fluorescence microscope (Leica, DM500); gradient PCR instrument (Bio-rad company in the United States); 7500 F real-time fluorescence quantitative PCR instrument (ABI company in the United States); electrophoresis instrument electrophoresis system (Shanghai Tanon Bio).

### 2.2. Method

#### 2.2.1. Cell proliferation detection

The cells were seeded in 96 well plates containing 100uL medium at the density of 4000 cells per well. After 24 hours, the final concentrations of 0, 200, 400 and 800 μm of ropivacaine were added to DMED, with 100 μL DMEM as negative control, three multiple holes were set and three independent experiments were carried out. After 48 and 72 h, MTT assay was used to detect cell proliferation. The detection wavelength was 562 nm with the reference wavelength at 630 nm.

#### 2.2.3. Cell scratch assay

Scratch test was performed to observe the cell migration ability. First, a marker pen was used to draw a horizontal line with a ruler on the back of the 6-hole plate and the line evenly crossed the hole. Each hole crosses at least five lines. The cells were added to the wells and after overnight cultivation, a ruler was used to scratch the horizontal line perpendicular to the back with the pipette tip. The cells were washed 3 times with PBS to remove the underlined cells, and serum-free medium was added. Incubated in 37°C incubator with 5% CO_2_. Samples were observed and photographed at 24, 48 h.

#### 2.2.4. Cell invasion experiment

To test whether ITGB1 inhibited migration of HCT116 and SW620 cells, Matrigel invasion test was used after 48 h transfection. 10^4^ cells/well were seeded in 200 μL of fetal bovine serum-free medium precoated with Matrigel in the upper chamber. Fill the lower well with 1 mL of completed DMEM medium to attract cell invasion. After incubation for 24 h, the cells on the surface of the membrane were fixed with 4% paraformaldehyde, stained with crystal violet, and observed under a microscope. A total of five random high-power microscopic fields (×200) were taken for each filter, and the number of cells was directly counted.

#### 2.2.5. DAPI nuclear staining

Cells were seeded on the 12 well plate with an initial density of 5 × 10^5^ cells/well, and crawling culture on the slide. After adhesion overnight, cells were treated with 0, 200, 400, and 800 μM ropivacaine, respectively. After 6 h, the cells on coverslip were washed with PBS, and stained with 1 μg/mL DAPI for 30 s. After being washed with pure water and fixed with fluorescent anti-quencher, the cells were imaged with a 400× fluorescence microscope.

#### 2.2.6. Apoptosis analysis

Cells (3.5–5 × 10^5^) were seeded in 6-well plates for cell culture and adherent overnight. The cells were cultured with 0, 200, 400, 800 μM ropivacaine. Standard growth medium was used for negative control. After incubating for 24 h, the supernatant was poured out from the cells to preserve the floating cells. Adherent cells were washed with PBS and digested with 0.25% trypsin; 5 μL FITC annexin and 5 μL propidium iodide (PI) were added to 100 μL cell suspension containing 10^5^ cells. The mixture was evenly mixed and incubated at room temperature for 15 minutes. After adding 400 μL binding buffer, the cells were analyzed by flow cytometry. All experiments were repeated three times.

#### 2.2.7. RT-PCR

The total RNA was extracted from the cells treated with 0, 800 μM ropivacaine. The cDNA was used as a template in SYBR Green real-time PCR Master Mix (Invitrogen, Carlsbad CA). The qPCR reaction was performed by ABI 7500 PCR system. The forward sequence of human ITGB1 primer was 5ʹ-TGATTGGCTGGAGGAATGTTA-3ʹ and the reverse sequence was 5ʹ-GTTTCTGGACAAGGTGAGCAA-3ʹ. The forward sequence of the control GAPDH primer was 5ʹ-AGAAGGCTGGGCTAATTTG-3ʹ and the reverse sequence was 5ʹ-AGGGGCCATCCACAGTCTTC-3ʹ. 2^−ΔΔCt^ method was used to calculate the expression level of ITGB1 gene.

#### 2.2.8. Western blot

Western blot analysis was performed on the lysates of cells treated with 0, 8 μM ropivacaine. After SDS-PAGE electrophoresis, the extracted cell proteins were transferred to PVDF membrane and blocked with 5% skim milk containing 0.05% Tween. The following rabbit polyclonal antibodies were used to Western blot the membrane: anti-ITGB1/Bax/caspase-3/a-caspase-3/caspase-9/a-caspase-9/FAK/ERK/AKT/p-FAK/p-ERK/p-AKT/GAPDH. They were washed in Tris buffer containing Tween-20 for 3 times, incubated with goat anti-rabbit secondary antibody at a dilution of 1:10,000 at room temperature for 1 h, and then washed with TBST. Finally, scanning with an ultraviolet imaging system.

#### 2.2.9. Cellular immunofluorescence

SW620 and HCT116 cells were inoculated in 6-well plate medium containing coverslips for cell crawling culture. After 24 hours of culture, 800 μM ropivacaine was added. The standard cell culture medium was used as a negative control. Cells were fixed with 4% paraformaldehyde after 8 h incubation in 5% CO2 37°C incubator. The rabbit anti-ITGB1 antibody was diluted with 1:1000 for 4°C overnight and then incubated with fluorescent secondary antibody for 1 h. Leica fluorescence microscope was used to observe and photograph.

#### 2.2.10. Drug target prediction

Structure and SMILE of ropivacaine were downloaded from NCBI PubChem, and the Similarity ensemble approach (SEA) database (http://sea.bkslab.org/) to predict potential target genes. Use the Genecards (https://www.genecards.org/) database to search with ‘colorectal cancer’ as the keyword to obtain disease targets. (The related documents are shown in the supplementary materials1, 2, 3.) Then, the gene cluster from the two databases are intersected, and then the potential binding targets of ropivacaine are screened based on experience and literature.

#### 2.2.11. Molecular docking

Download the structure of compound ropivacaine from the PubChem database (https://pubchem.ncbi.nlm.nih.gov/), and optimize the compound structure in the MMFF94 force field of OpenBabel 2.4.1. The three-dimensional structure of ITGB1 is downloaded from RCSB Protein Data Bank (http://www.rcsb.org/) to obtain the three-dimensional structure of ITGB1 (PDB ID: 4WK0). Use AutodockTools1.5.6 to search and define the compound’s rotatable bond; Repair the ITGB1 protein structure by deleting residues and terminal residues on the polypeptide chain, removing water molecules, adding hydrogen atoms, adding charges, and save it in PDBQT format. Autodock vina 1.1.2 was used for molecular docking research. We used POCASA 1.1 (http://g6altair.sci.hokudai.ac.jp/g6/service/pocasa/) to make a pocket prediction of the protein, thus determining the coordinates of the active site of ITGB1: center_x = 19.5, center_y = 13.0, Center_z = −61.3; size_x = 36.2, size_y = 27.0, size_z = 23.8, and other parameters use the default values. The conformation with the lowest docking energy is selected for docking mode analysis, and Pymol is used for mapping (Find in the supplementary material).

#### 2.2.12. Statistics

The data were presented as the mean ± standard deviation. The statistical analysis was performed using Student’s *t*-test (for two experimental groups). After statistical processing, the significance was set at *P* < 0.05. Statistical significance was determined using Student’s *t*-test and *F-*test (F-test was used for the statistics of apoptosis level). The Graphpad Prism 8.0 software was used for the visualization of the statistical.

## 3. Results

### 3.1. Ropivacaine inhibited the proliferation, migration and invasion of HCT116 and SW620 cells

**Figure 1.**
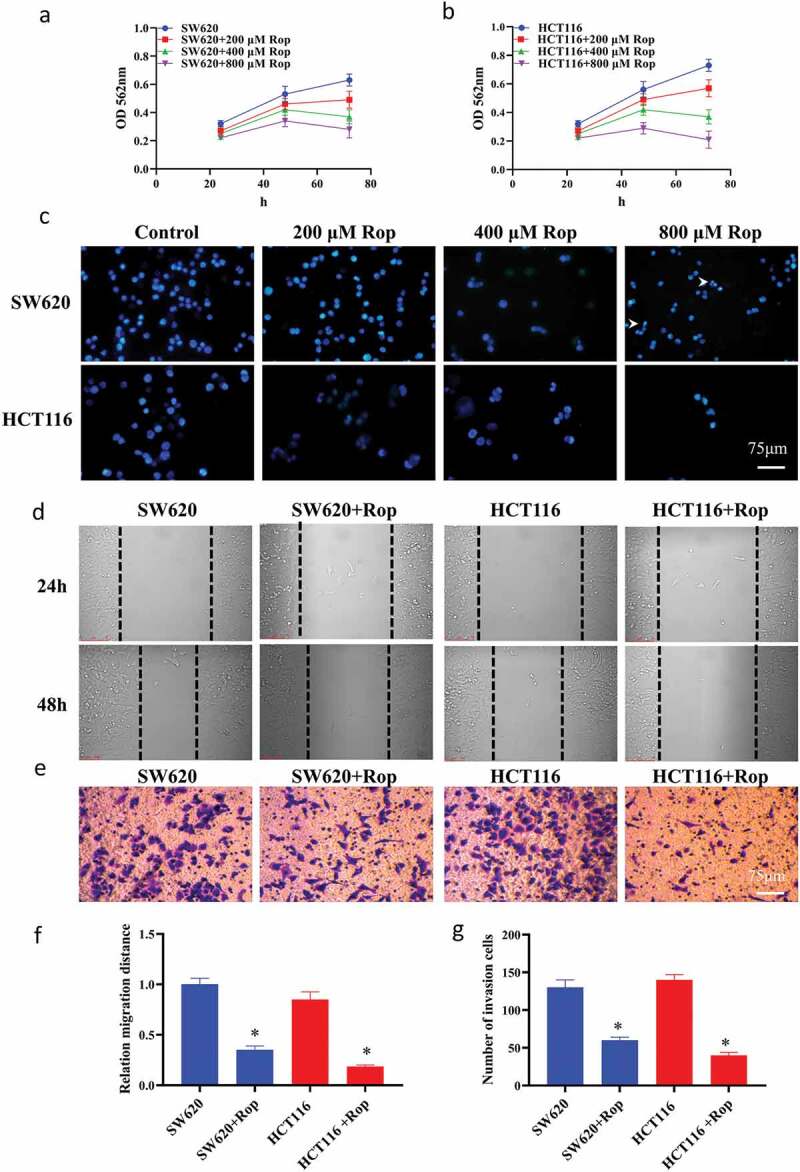
Ropivacaine inhibits the proliferation and migration of HCT116 and SW620 cells, and the invasion ability. (a). SW620 and different doses of ropivacaine after treatment, and the results of MTT detection were expressed by absorbance values at 562 nm; (b). HCT116 and different doses of ropivacaine after treatment, the results of MTT detection were expressed by absorbance values at 562 nm; (c). The result of nuclear staining of two colon cancer cells treated with different doses by DAPI; (d). Changes in cell migration within 24 h; (e). Transwell experiment results; (f). Quantitative statistical results of cell migration experiments; **G**. Transwell experiment to quantify statistical results. ‘*’ indicates a significant difference compared with the control group (*P* < 0.05). ‘**’ indicates a significant difference (*P* < 0.01), the same below

In this study, HCT116 and SW620 cells were treated with different concentrations of ropivacaine (0, 200, 400, 800 μM), and the growth of HCT116 and SW620 cells after 48 and 72 h was detected by MTT method. The results showed that ropivacaine had a dose-dependent effect on the growth of colorectal cancer cells, and the inhibitory effect of 800 μM ropivacaine on colon cancer cells was the most significant (*P* < 0.05) (Figure 1(a,b). DAPI staining showed (Figure 1(c)) that after the number of apoptotic bodies increased significantly with the increase of ropivacaine concentration, especially in HCT116 cells. When the concentration of ropivacaine was 800 μM, HCT116 cells basically floated, the cell state was poor, and the cell adhesion was the least. Therefore, 800 μM ropivacaine was determined as the drug concentration for subsequent experiments. The results of the scratch test showed (Figure 1(d,f)) that when the concentration of ropivacaine was 800 μM, the migration distance of colon cancer cells was significantly inhibited within 24 h, and there was a significant difference between the migration distance of colon cancer cells and that of normal colon cancer cells (*P* < 0.05). The invasion experiment results showed (Figure 1(e,g)) that after treatment with ropivacaine, the invasion number of colon cancer cells was significantly reduced (*P* < 0.05).

### 3.2. Ropivacaine promoted apoptosis of colon cancer cells

**Figure 2.**
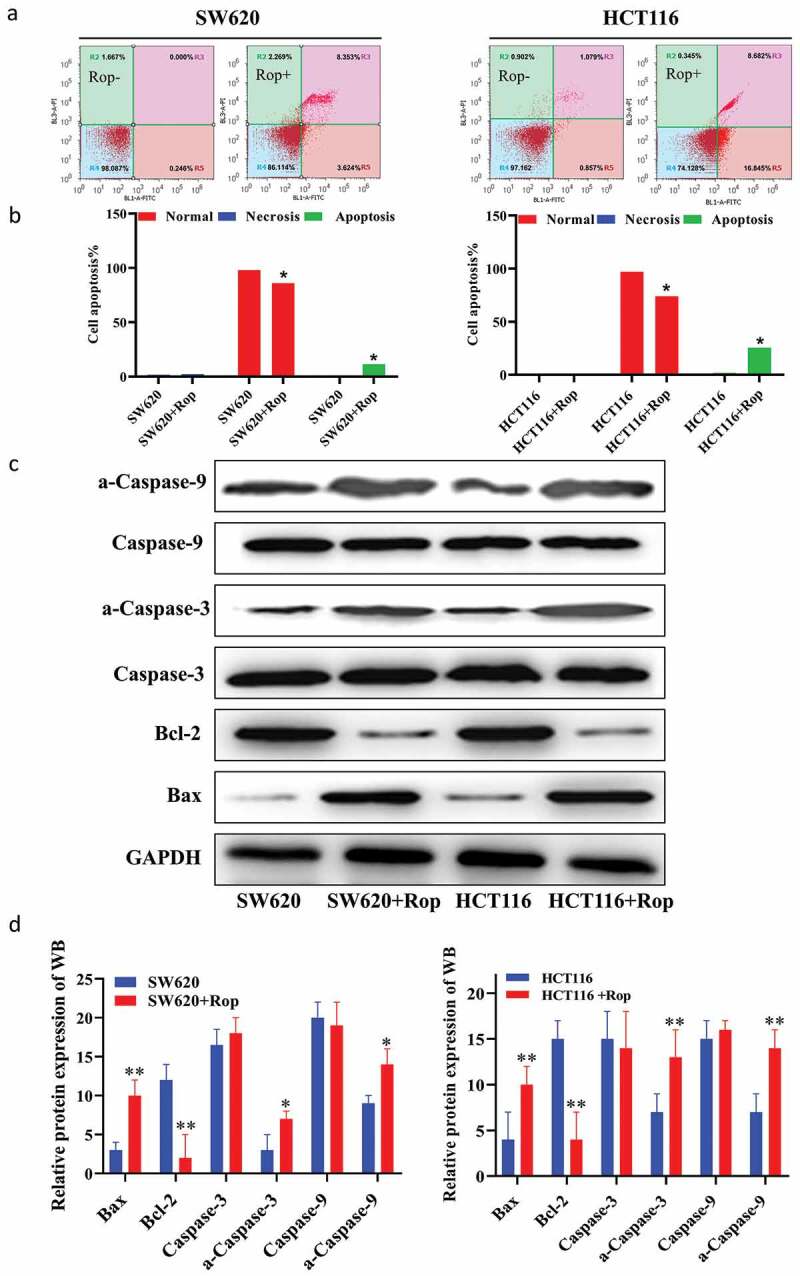
Ropivacaine promoted apoptosis of colorectal cancer cells. (a). Flow cytometry to detect apoptosis results; (b). Quantitative graph of apoptosis ratio by flow cytometry to detect apoptosis results; (c). Immunoblotting to detect the expression of apoptosis-related proteins; (d). Quantification of immunoblotting results

In order to explore the effect of ropivacaine on apoptosis of colon cancer cells, HCT116 and SW620 cells were treated with 800 μM ropivacaine for 24 h and then detected the apoptosis by flow cytometry. The results showed that compared with normal colon cancer cells, the percentage of apoptosis of colon cancer cells increased significantly after adding ropivacaine (Figure 2(b) for quantification diagram). Compared with normal colon cancer cells, ropivacaine could up-regulate the expression of Bax, caspase-3, a-caspase-3, caspase-9, a-caspase-9, and down-regulate the expression of bcl-2. There was a significant difference between the two groups (Figure 2(c,d)).

### 3.3. Targeting relationship between ropivacaine and ITGB1 protein

**Figure 3.**
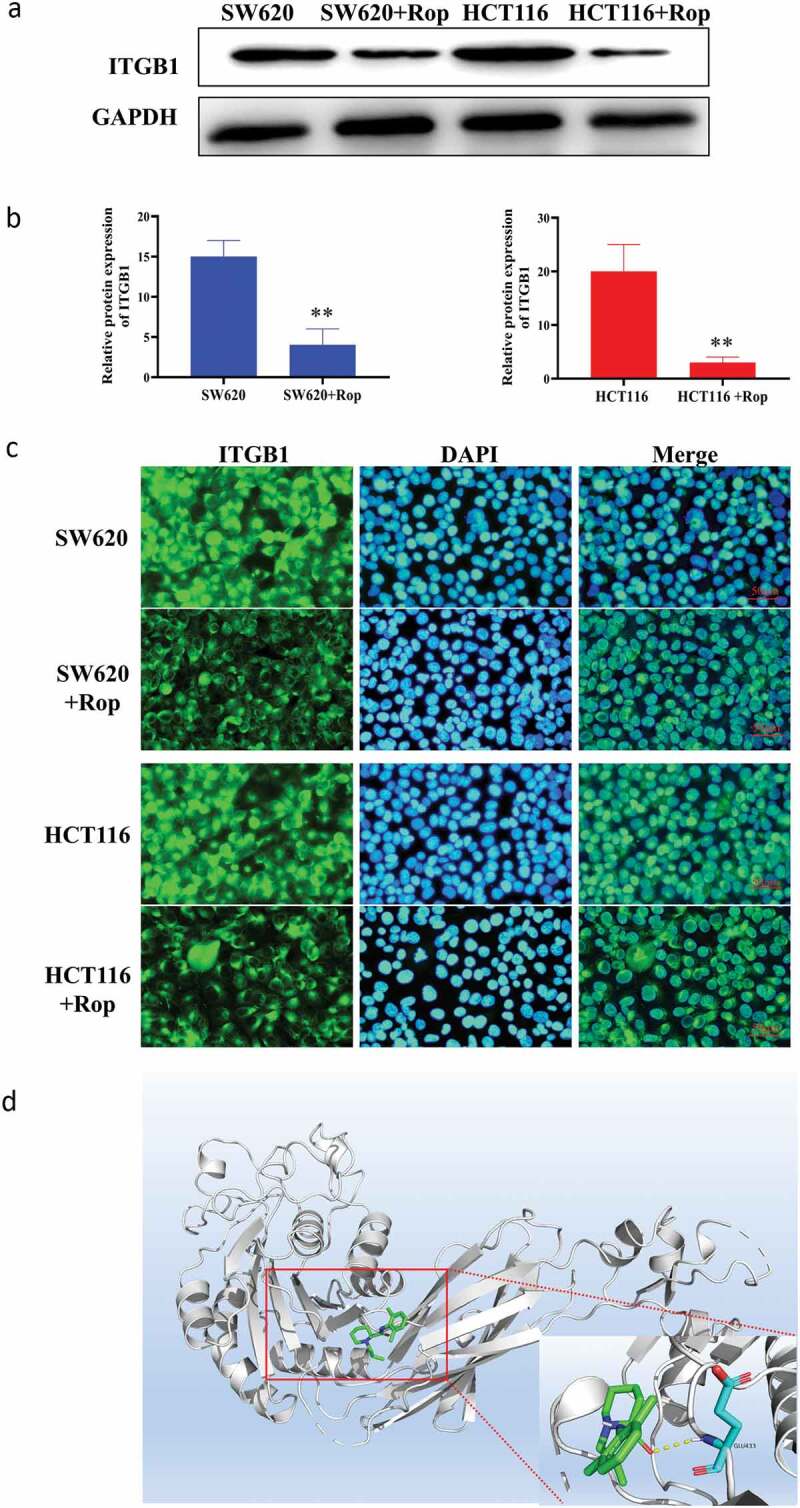
Targeting relationship between ropivacaine and ITGB1 protein. (a). Immunoblotting to detect the expression of ITGB1 protein; (b). Quantitative graph of immunoblotting results; (c). Immunofluorescence detection of the distribution and expression of ITGB1 protein in cells; (d). Bioinformatics predicts the steric binding site of ropivacaine and ITGB1 protein

**Figure 4.**
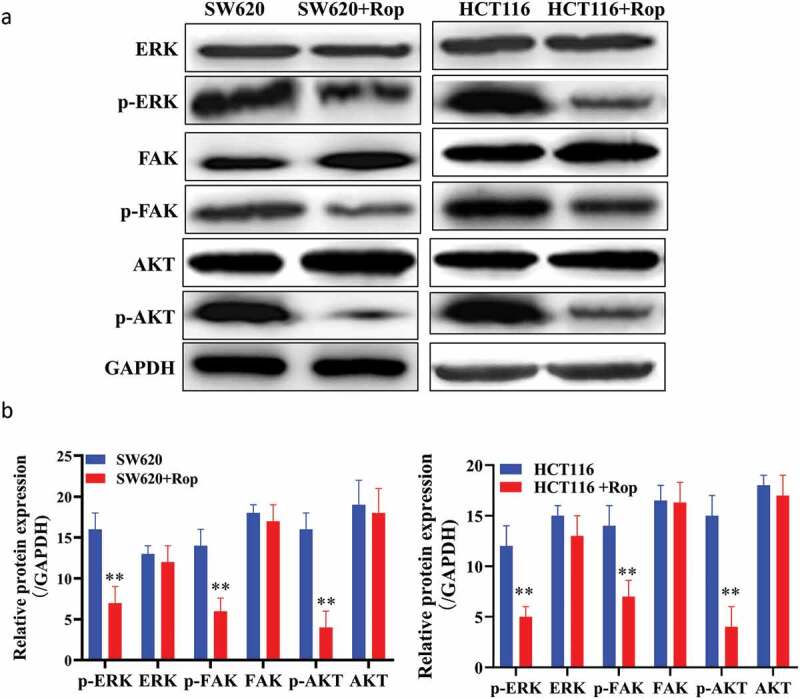
Ropivacaine inhibited the expression of ITGB1 protein and affected the activation of its downstream signaling pathway. (a). Western blot detection of the downstream pathway of ITGB1 protein; (b). Quantification of Western blot detection results

Online prediction websites were used to predict the target gene of ropivacaine. As shown in Figure 3(d), ropivacaine can form hydrogen bond with GLU433 in ITGB1 protein, and then form stable complex with ITGB1. Western blot results showed that the expression of ITGB1 protein in HCT116 and SW620 cells decreased significantly after adding ropivacaine (Figure 3(a,b)). Immunofluorescence results showed that ITGB1 was highly expressed in HCT116 and SW620 cells. After treatment with ropivacaine, the positive rate of ITGB1 protein was significantly decreased, indicating that ITGB1 protein was inhibited (Figure 3(c)). Furthermore, the downstream pathway of ITGB1 protein was further studied, and the phosphorylation of FAK/ERK/Akt protein and its protein was detected by Western blot. It was found that the downstream pathway proteins of ITGB1 protein were also inhibited, and the phosphorylation degree was increased (Figure 4(a,b)). These results indicate that ropivacaine may exert its biological function by targeting ITGB1 and then influencing its downstream signaling pathway.

## 4. Discussion

The aim of this study was to investigate the effects of ropivacaine on proliferation, cell cycle, and apoptosis of colon cancer cells. Objective to investigate the effect of ropivacaine on the proliferation and migration of colon cancer cells by targeting ITGB1. Colorectal cancer is a disease that affects millions of people worldwide [1]. Previous studies have confirmed that ropivacaine plays a key role in the progression of colon cancer [13,19]. Bundscherer et al. [19]found that ropivacaine, bupivacaine, and sufentanil could not change the apoptosis rate and cell cycle distribution of HT-29, SW480, PaTu 8988 t and PANC 1 cancer cells at clinical used concentrations. Only higher concentrations of ropivacaine or bupivacaine were anti-proliferative. The results of Siekmann et al. [20] showed that both lidocaine and ropivacaine had anti-proliferative effects on colon cancer cell lines after high concentrations and long-term exposure to LA in vitro. In this study, we used different concentrations of ropivacaine for long-term exposure in vitro, and all showed anti-proliferative effects on HCT116 and SW620 colon cancer cells. Among them, ropivacaine at low concentrations (1–10 μM) will not significantly reduce cell survival of human colon cancer cell lines. The viability of colon cancer cells was more significantly dependent on the reduction of ropivacaine at a concentration of 200 μM-1 M. Our results are consistent with Le Gac’s research on liver cancer cell lines [21].

Baptista-Hon et al. [13] detected that the expression of subtype Nav1.5 in colon cancer tissue was significantly increased compared with normal colon tissue. In HT 29, SW480, and SW620, knocking out Nav1.5 in vitro inhibited the invasion of colon cancer cells. Ropivacaine has a strong Nav1.5 inhibition effect on SW620 colon cancer cells and has a certain anti-cancer effect. β1 integrin (ITGB1) is the integrin protein mainly expressed in normal cells and tumor-related cells. It was usually upregulated in human malignancies and was involved in many developmental processes, such as tumor progression and metastasis. Song et al. [22] showed that the upregulation of ITGB1 significantly increased the proliferation and cell invasion ability of HT29 cells in vitro. It was also found that ITGB1 was an effective growth factor in a xenograft tumor mouse model. In recent years, there has been increasing evidence that voltage-gated sodium channels are expressed in human malignant tumor cells including breast cancer, colon cancer, and prostate cancer. It seems to be related to aggressive and metastatic phenotypes. However, whether ropivacaine inhibited cell proliferation and migration through ITGB1 expression in colon cancer cells remains unclear. First, MTT assay proved that ropivacaine has an apparent inhibitory effect on colon cancer cells. A dose-dependent correlation, especially the inhibitory effect of 800 μ m ropivacaine, is the most obvious. DAPI staining from the nuclear morphology of colon cancer cell apoptosis, nuclear pyknosis significantly reduced survival cells. Cell migration and invasion experiments directly reflect ropivacaine’s ability to inhibit the migration and invasion of colon cancer cells. These phenomena prove that ropivacaine has research value in the treatment of colon cancer. After identifying the phenomenon. We started the follow-up study. The target protein of ropivacaine was predicted by bioinformatics. The binding energy of itgb1 was found to be 5.9 kcal/mol, suggesting that there may be binding between them, which provides the basis for further study on ropivacaine’s targeting effect on itgb1. Cell immunofluorescence, RT-PCR, and Western blot analysis showed that ropivacaine inhibited the expression of itgb1 and affected the phosphorylation of Akt/FAK/ERK, the downstream pathway of itgb1. This study is the first time to show that ropivacaine can significantly reduce the expression of itgb1 in colon cancer cells. These data indicate that ropivacaine inhibits the occurrence and development of colorectal cancer by inhibiting the expression of itgb1 and phosphorylation of its downstream pathway, thus playing a role in tumor inhibition.

## 5. Conclusion

This study showed that under the action of ropivacaine, the expression of ITGB1 in HCT116 and SW620 cells was significantly reduced, and ropivacaine can inhibit the proliferation, migration, and invasion of colorectal cancer cells. In addition, we confirmed for the first time that ropivacaine regulates the biological function of colon cancer cells through ITGB1.

## Supplementary Material

Supplemental Material

Supplemental Material

Supplemental Material

## Data Availability

The datasets used or/and analyzed during the current study are available from the corresponding author on reasonable request.

## Highlight

- Ropivacaine inhibited the proliferation, migration, and invasion of HCT116 and SW620 cells.
- Ropivacaine promoted apoptosis of colon cancer cells.
- Ropivacaine inhibits the growth of colon cancer cells by targeting ITGB1.

## References

[1] Din FV, Theodoratou E, Farrington SM, et al. Effect of aspirin and NSAIDs on risk and survival from colorectal cancer. Gut. 2010;59(12):1670–1679. . 20844293 10.1136/gut.2009.203000

[2] Peto J. Cancer epidemiology in the last century and the next decade. Nature. 2001;411(6835):390–395. 11357148 10.1038/35077256

[3] Tsukuma H, Ajiki W. Descriptive epidemiology of colorectal cancer–international comparison. Nihon Rinsho. 2003;61(Suppl 7):25–30. 14574850

[4] Mou X, Chen L, Liu F, et al. Prevalence of JC virus in Chinese patients with colorectal cancer. PLoS One. 2012;7(5):e35900. . 22606241 10.1371/journal.pone.0035900 PMC3350510

[5] Gottschalk A, Sharma S, Ford J, et al. Review article: the role of the perioperative period in recurrence after cancer surgery. Anesth Analg. 2010;110(6):1636–1643. 20435944 10.1213/ANE.0b013e3181de0ab6

[6] Goldfarb Y, Ben-Eliyahu S. Surgery as a risk factor for breast cancer recurrence and metastasis: mediating mechanisms and clinical prophylactic approaches. Breast Dis. 2006;26:99–114. 17473369 10.3233/bd-2007-26109

[7] Bharati SJ, Chowdhury T, Bergese SD, et al. Anesthetics impact on cancer recurrence: what do we know? J Cancer Res Ther. 2016;12(2):464–468. 27461594 10.4103/0973-1482.148670

[8] Ni J, Xie T, Xiao M, et al. Amide-linked local anesthetics preferentially target leukemia stem cell through inhibition of Wnt/β-catenin. Biochem Biophys Res Commun. 2018;503(2):956–962. 29932919 10.1016/j.bbrc.2018.06.102

[9] Beilin B, Shavit Y, Trabekin E, et al. The effects of postoperative pain management on immune response to surgery. Anesth Analg. 2003;97(3):822–827. . 12933409 10.1213/01.ANE.0000078586.82810.3B

[10] Kurosawa S. Anesthesia in patients with cancer disorders. Curr Opin Anaesthesiol. 2012;25(3):376–384. 22450698 10.1097/ACO.0b013e328352b4a8

[11] Chang YC, Liu CL, Chen MJ, et al. Local anesthetics induce apoptosis in human breast tumor cells. Anesth Analg. 2014;118(1):116–124. . 24247230 10.1213/ANE.0b013e3182a94479

[12] Lirk P, Berger R, Hollmann MW, et al. Lidocaine time- and dose-dependently demethylates deoxyribonucleic acid in breast cancer cell lines in vitro. Br J Anaesth. 2012;109(2):200–207. 22542536 10.1093/bja/aes128

[13] Baptista-Hon DT, Robertson FM, Robertson GB, et al. Potent inhibition by ropivacaine of metastatic colon cancer SW620 cell invasion and NaV1.5 channel function. Br J Anaesth. 2014;113(Suppl 1):i39–i48. . 24852501 10.1093/bja/aeu104

[14] Perez-Castro R, Patel S, Garavito-Aguilar ZV, et al. Cytotoxicity of local anesthetics in human neuronal cells. Anesth Analg. 2009;108(3):997–1007. . 19224816 10.1213/ane.0b013e31819385e1

[15] Lucchinetti E, Awad AE, Rahman M, et al. Antiproliferative effects of local anesthetics on mesenchymal stem cells: potential implications for tumor spreading and wound healing. Anesthesiology. 2012;116(4):841–856 . 22343474 10.1097/ALN.0b013e31824babfe

[16] Bianconi D, Unseld M, Prager GW. Integrins in the spotlight of cancer. Int J Mol Sci. 2016;17(12). DOI:10.3390/ijms17122037 PMC5187837 27929432

[17] Brakebusch C, Fässler R. beta 1 integrin function in vivo: adhesion, migration and more. Cancer Metastasis Rev. 2005;24(3):403–411. 16258728 10.1007/s10555-005-5132-5

[18] Arao S, Masumoto A, Otsuki M. Beta1 integrins play an essential role in adhesion and invasion of pancreatic carcinoma cells. Pancreas. 2000;20(2):129–137. 10707927 10.1097/00006676-200003000-00004

[19] Bundscherer A, Malsy M, Gebhardt K, et al. Effects of ropivacaine, bupivacaine and sufentanil in colon and pancreatic cancer cells in vitro. Pharmacol Res. 2015;95-96:126–131. 25839130 10.1016/j.phrs.2015.03.017

[20] Siekmann W, Tina E, Von Sydow AK, et al. Effect of lidocaine and ropivacaine on primary (SW480) and metastatic (SW620) colon cancer cell lines. Oncol Lett. 2019;18(1):395–401. 31497075 10.3892/ol.2019.10332 PMC6728124

[21] Le Gac G, Angenard G, Clément B, et al. Local anesthetics inhibit the growth of human hepatocellular carcinoma cells. Anesth Analg. 2017;125(5):1600–1609. 28857796 10.1213/ANE.0000000000002429

[22] Song J, Zhang J, Wang J, et al. β1 integrin mediates colorectal cancer cell proliferation and migration through regulation of the Hedgehog pathway. Tumour Biol. 2015;36(3):2013–2021. 25387809 10.1007/s13277-014-2808-x

