# Supplementary figures and images for: Ropivacaine inhibits the proliferation and migration of colorectal cancer cells through ITGB1
Source: Bioengineered. 2020 Dec 21;12(1):44–53. doi: 10.1080/21655979.2020.1857120 (PMC8806321; doi:10.1080/21655979.2020.1857120)

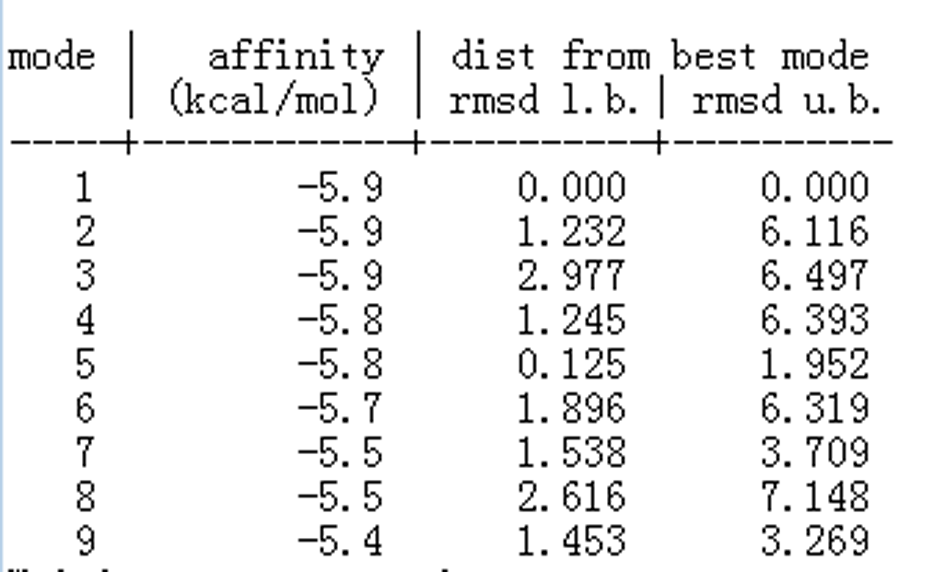

Supplement: Supplemental Material [file KBIE_A_1857120_SM0947.tif]
